# Focused and distributed: Diverse attentional profiles in pigeon category learning

**DOI:** 10.3758/s13420-026-00706-6

**Published:** 2026-01-27

**Authors:** Leyre Castro, Ananya Albrecht-Buehler, Edward A. Wasserman

**Affiliations:** Department of Psychological and Brain Sciences, The University of Iowa, Iowa City, IA 52242 USA

**Keywords:** Categorization, Attention, Individual differences, Animal cognition, Pigeons

## Abstract

Attention to relevant information is necessary to solve a categorization task. Prior studies monitoring peck location indicated that pigeons narrowly focus their attention on the relevant features of a category exemplar, whereas computational modeling suggested that pigeons’ attention can be widely distributed. To help resolve these apparently contradictory results, we deployed both strategies – peck tracking and computational modeling – in the same experiment. Pigeons’ peck-tracking scores neatly aligned with their category choices and with the results of the computational modeling. In addition, pigeons displayed distinctive learning and attentional patterns during training and testing. Half of our pigeons showed preferential, even exclusive, use of the most relevant category features, but the other half were inclined to use several of the available features. These results highlight the importance of combining an individual differences approach along with the pursuit of general cognitive principles.

**Supplementary Information:**

The online version contains supplementary material available at https://doi.org/10.3758/s13420-026-00706-6.

## Introduction

Learning to classify objects and events requires noticing and processing the elements that are characteristic of those objects and events. A round shape is relevant to determining that an object is a ball; so, organisms need to attend to shape if they are to distinguish, for example, balls from bricks. Thus, attention to relevant information is necessary to successfully perform a categorization task.

Theories of animal associative learning (e.g., George & Pearce, 2012; Mackintosh, 1965, 1975) as well as theories of human category learning (Kruschke, 1992; Nosofsky, 1986, 1992; Smith & Minda, 1998) consider attention to be a necessary component of the learning process. Moreover, they concur that attention tends to be distributed among multiple stimulus features in the initial stages of learning, but that it converges on the most relevant features as learning proceeds. Considerable research has shown that pigeons are exceptional categorizers of complex visual stimuli (e.g., Cook, Wright, & Kendrick, 2019; Lazareva & Wasserman, 2010; Pusch, Clark, Rose, & Güntürkün, 2023). It is therefore logical to assume that they must pay attention to the information that is relevant to solving a categorization task, whether this information is a single, specific feature (e.g., Wasserman & Castro, 2021a, 2021b) or a stimulus dimension (e.g., Qadri, Ashby, Smith, & Cook, 2019).

Attention cannot be directly observed, so it must be inferred from an organism’s behavior. Just as eye-tracking is considered to be a proxy for attention in human categorization (Rehder & Hoffman, 2005b), *peck tracking* could be considered to be a suitable proxy for attention in pigeons. On that premise, touchscreen technology has been used to track pigeons’ peck location within complex images. Dittrich, Rose, Buschmann, Bourdonnais, and Güntürkün (2010) tracked pigeons’ peck locations while learning a go/no-go task that required discrimination between images in which human figures were present or absent. As pigeons’ discriminative performance improved, they focused their pecks more on the areas occupied by the human figures, the distinctively diagnostic features of the pictorial stimuli.

In the same vein, Castro and Wasserman (2014) trained pigeons to classify stimuli from two different artificial categories, in which the exemplars contained both relevant and irrelevant features. When an exemplar was presented on the computer screen, pigeons had to peck it several times. We recorded the location of pigeons’ pecks in order to determine whether or not they selectively directed their pecks to the relevant features of the category exemplars. We found that, as pigeons’ categorization accuracy progressively increased, so too did their pecks to the relevant category features. Thus, as pigeons were learning to categorize the different category exemplars, they also learned to attend preferentially to their relevant features. This and subsequent studies using the peck-tracking technique (Castro & Wasserman, 2016, 2017; Castro, Remund-Wiger, & Wasserman, 2021; Sheridan, Castro, Fonseca, & Wasserman, 2019) provide strong evidence that pigeons predominantly track the relevant features that predict category membership.

But categories may contain information that is relevant to different degrees or at different levels. A considerable amount of research on pigeon’s categorization has examined whether the birds are more likely to attend to the elements contained in a larger, more complex stimulus, or to the stimulus as a whole; that is, whether pigeons attend to local or global features of the stimuli (e.g., Aust & Braunöder, 2015; Cerella, 1977; Clark & Colombo, 2022; Cook, 2001; Fremouw, Herbranson, & Shimp, 1998; Goto, Wills, & Lea, 2004). Despite initial conclusions that pigeons were primarily processors of local information, it is currently accepted that pigeons adapt their perceptual strategies based on task demands and environmental factors (Clark & Colombo, 2022; Huber & Aust, 2012). Rather than being locked into a single local or global processing mode, pigeons can shift their attention between different levels of visual analysis from local details to global patterns (Aust & Braunöder, 2015; Cook, 2001) and from individual elements to configural wholes (Aust & Huber, 2003) in order to optimize performance (Huber & Aust, 2012).

Thus, pigeons’ attention may also be widely distributed among multiple features or dimensions, rather than being narrowly focused on the most diagnostic information. In our peck-tracking studies, the relevant features were always perfect predictors of the category, whereas the other features were unrelated to any of the categories. It is possible that, if other features with some but less predictive value were available, then pigeons might attend in a more global or diffuse way. To investigate this issue, Castro et al. (2020) gave pigeons a categorization task in which exemplars contained a single deterministic feature that perfectly predicted category membership accompanied by six other features that only probabilistically predicted category membership. In this case, the task could be learned on the basis of either one *deterministic* feature (encouraging selective, focused attention) or multiple *probabilistic* features (encouraging distributed attention). In that project, we measured pigeons’ categorization responses, but not their peck tracking.^1^

Pigeons mastered this categorization task. To better understand how they did so, we tested them with new category exemplars in which the deterministic feature was absent, but all the probabilistic features were present, as well as with new category exemplars in which the deterministic feature was present, but one or more of the probabilistic features were absent. Then, we used Nosofsky’s (1986, 1988, 1992) Generalized Context Model (GCM) to estimate the attentional weights that best accounted for the pigeons’ categorization responses (we also use GCM to analyze the results of our current experiment; further details about the model are included in the *Method* section). Following this computational strategy, Castro et al. (2020) found that pigeons distributed their attention among several features. Pigeons did not appear to strongly focus attention on only the deterministic feature. Rather, pigeons tended to distribute their attention among the various features of the category exemplars.

**Fig. 1:**
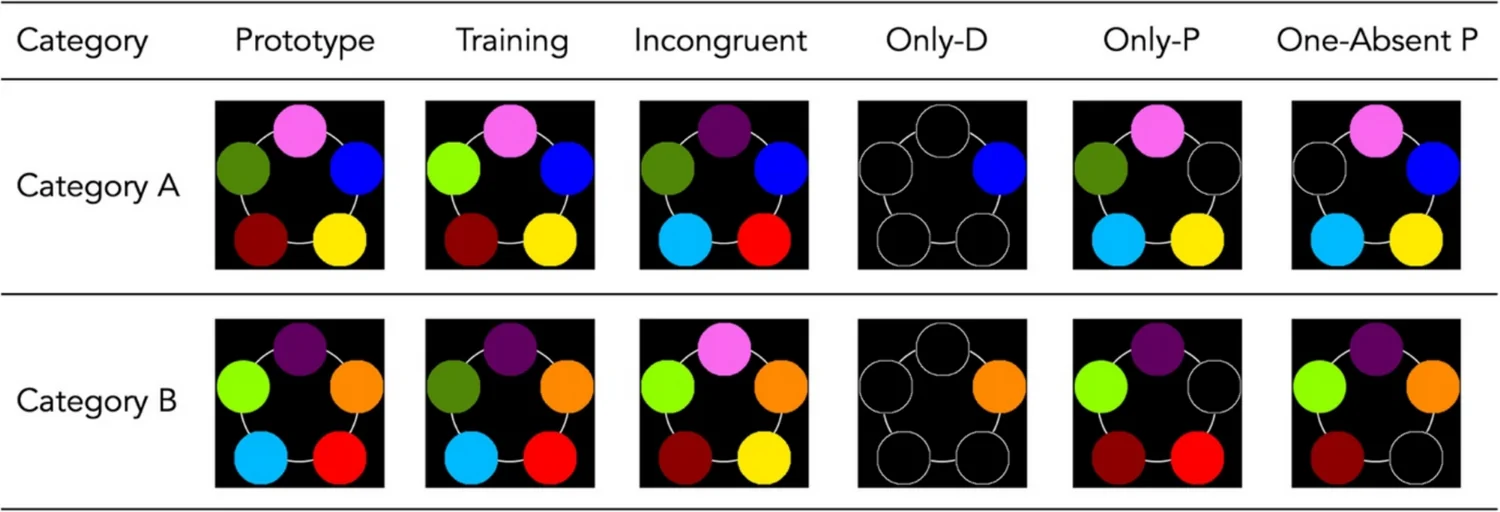
Examples of the training and testing category exemplars used. Each row depicts stimuli within a category. Here, the deterministic feature for Category A is the dark blue circle on the right side of the stimulus, whereas the deterministic feature for Category B is the orange circle in the same location. Training stimuli were presented in both training and testing phases. Prototype, Incongruent, Only-P, Only-D, and One-Absent-P stimuli were presented only in the testing phase. For absent features, a black circle with a white outline was presented

In summary, studies using peck tracking suggested that pigeons’ attention was effectively focused on the relevant features of a category exemplar (Castro & Wasserman, 2014, 2016, 2017; Castro, Remund-Wiger, & Wasserman, 2021; Dittrich et al., 2010; Sheridan et al., 2019), whereas computational modeling indicated that pigeons’ attention was quite distributed (Castro et al., 2020). To help resolve these apparently contradictory results, and to better characterize the pigeons’ category learning behavior, in the present study we deployed both techniques – peck tracking and computational modeling – in the same experiment with the same visual stimuli. We used the same stimuli as Castro et al. (2020). However, to facilitate the recording of peck tracking, we made the category exemplars slightly larger, and we reduced the number of features from seven to five: one feature was deterministic and the other four were probabilistic (see Fig. 1). Note that, compared to our prior peck-tracking studies, here there are *no* totally irrelevant features; the probabilistic features still have some predictive, albeit low, value.

In addition, unlike all of our prior peck-tracking studies, we did not require the pigeons to peck within the areas occupied by the features of the category exemplar; the birds were free to peck anywhere within the category exemplars (approximately half of the area of a given exemplar was occupied by the stimulus features, and the remaining area was black) in order to complete their observing response requirement. This way, we avoided leading the pigeons’ attention to the features of each exemplar and, consequently, we could collect clearer evidence of their natural observing behavior.

An important advantage of the peck-tracking technique is that it provides a continuous measure that allows us to document the progress of attention. The computer-modeling strategy, on the other hand, needs to be performed at a specific point in time, so it provides a more static measure of attention. Similar to Castro et al. (2020), here we conducted a final testing phase after the pigeons had fully mastered the categorization task, and we computed the entropy of pigeons’ attentional profiles at that point. But we also included intermediate tests after the birds reached intermediate levels of accuracy, so we could compute their attentional profiles throughout the learning process.

Recall that in prior experiments we had found that pigeons’ tracking of relevant information increased throughout training. This relevant-information tracking may have been facilitated by the fact that the other features were completely irrelevant. Here, with stimuli that also contain features with low, imperfect predictive values, we hoped to replicate pigeons’ tracking of the most relevant information – in this case, the deterministic feature – although the stimulus structure allowed them to solve the task even without attending to this deterministic feature (attending to the three relevant probabilistic features could allow the birds to reach accuracy as high as accuracy using the deterministic feature). In addition, we expected the attentional profiles derived from the intermediate tests to be more distributed at the beginning of training and more focused as learning progressed.

## Method

### Subjects

The subjects were six homing pigeons (*Columba livia*) – three females (17R, 48 W, and 56Y) and three males (30Y, 37 W, and 56B) – that were maintained at 85% of their free-feeding weights by controlled daily feedings. The pigeons had served in unrelated studies prior to the present project. All procedures were approved by the Institutional Animal Care and Use Committee at The University of Iowa.

### Apparatus

The experiment used four 36 × 36 × 41 cm operant conditioning chambers detailed by Gibson, Wasserman, Frei, and Miller (2004). The chambers were in a dark room with continuous white noise. Each chamber was equipped with a 15-in. LCD monitor located behind an AccuTouch^®^ resistive touchscreen (Elo TouchSystems, Fremont, CA, USA). The portion of the screen that was viewable by the pigeons was 28.5 × 17.0 cm. Pecks to the touchscreen were processed by a serial controller board outside the box. A rotary dispenser delivered 45-mg pigeon pellets through a vinyl tube into a food cup located in the center of the rear wall opposite the touchscreen. Illumination during the experimental sessions was provided by a houselight mounted on the upper rear wall of the chamber. The pellet dispenser and houselight were controlled by a digital I/O interface board. Each chamber was controlled by its own Windows^®^ computer. The program to run the experiment was developed in MatLab^®^ with Psychtoolbox-3 extensions (Brainard, 1997; Pelli, 1997; http://psychtoolbox.org/).

### Stimuli

*Training stimuli.* A total of ten colored 2.7-cm diameter circles were used to create the different category training exemplars. These colors’ wavelengths were all readily discriminable by pigeons (Emmerton & Delius, 1980; Palacios & Varela, 1992).

Pigeons were trained on two categories: A and B. Each of the category exemplars contained five features forming a circle (see Fig. 1). The features were separated from one another, but were connected by a white line. Each exemplar occupied a 10-cm square area. Each category was derived from a prototype that differed in all five colors from the prototype of the other category; this prototype was *never* presented during training. In training, all exemplars contained one deterministic feature that perfectly distinguished the two categories; in addition, the exemplars contained three probabilistic features consistent with the corresponding prototype and one more feature consistent with the opposite prototype. For any given bird, each colored circle was always presented in the same location so that the total number of unique training exemplars was four for Category A and four for Category B.

To control for a possible preference in attending to certain areas of the stimulus array, the deterministic feature was placed in a different location for each of three sets. Two birds were trained on each set. Additionally, to control for the possibility that some color combinations were more discriminable than others, category exemplars in each sets contained features of different colors. Examples of training and testing exemplars from one of the sets are shown in Fig. 1. All training and testing exemplars for the three sets are available in the Open Science Framework (OSF) repository at https://osf.io/e35qv/?view_only=a9a9ad60883249b19fbc1859ae582e17

*Testing stimuli.* In testing, birds were presented with six types of stimuli: Prototype, Training, Incongruent, Only Deterministic (Only-D), Only Probabilistic (Only-P), and One Absent Probabilistic

(One-Absent-P). Training stimuli were used to assess learning of the trained categories. Incongruent stimuli had the deterministic feature from one category, but most of the probabilistic features consistent with the opposite category; they allowed us to determine whether the birds relied on the overall similarity of the exemplars or on the deterministic feature. Only-D stimuli contained only the deterministic feature, with empty circles replacing all probabilistic features; they allowed us to determine whether birds could rely on the deterministic feature when none of the probabilistic features were available. Only-P stimuli had the probabilistic features of one category and an empty circle replacing the deterministic feature; they allowed us to assess whether birds could rely on the probabilistic features when the deterministic feature was unavailable. In One-Absent-P stimuli, one of the probabilistic features congruent with the stimuli category was replaced by an empty circle to see if the replacement of just one relevant feature (as in Only-P stimuli) would produce a drop in performance. Finally, in Prototype stimuli, all probabilistic features belonged to the congruent category; they allowed us to see if the gain in probabilistic features from the correct category and/or the loss of probabilistic features from the incorrect category would produce a rise in performance.

### Procedure

*Acclimation.* All pigeons were shown all of the training stimuli in their assigned set and the two response buttons (2.3 x 6.0 cm rectangles filled with two distinctive black-and-white patterns) for three sessions. Each stimulus image was presented nine times, and each response button was presented 18 times, for a total of 108 trials. Training stimuli were presented in the middle of the screen, whereas the response buttons were presented 4.5 cm to the left and right of the area occupied by the stimulus. These placements corresponded with where these stimuli would later be displayed. The pigeons were required to peck each stimulus a number of times that increased in each session (gradually, from two to six pecks). After fulfilling the response requirement, one or two food pellets were randomly delivered. This phase allowed the birds to acclimate to the stimuli. After the third acclimation session, pigeons moved on to training.

*Training.* Each training session comprised 128 trials: half presented Category A exemplars and half presented Category B exemplars, in a random fashion. At the beginning of a trial, a 3.0-cm white square appeared in the center of the computer screen. After one peck anywhere on this white square, a category exemplar was displayed in the center of the screen. To progress, pigeons needed to satisfy an observing response requirement of *n* pecks *anywhere* within the area occupied by the category exemplar. We recorded the location of these pecks to determine whether the pigeons directed their pecks to the deterministic feature or to the probabilistic features or to the black areas around the features. The response requirement was increased by one after every training session until a maximum of six pecks, and then was maintained at six pecks for all subsequent sessions.

On completion of the observing response requirement, the response buttons appeared to the left and right of the category exemplar. The pigeons had to peck one of the two report buttons, depending on the category presented. If the choice was correct, then one or two food pellets were delivered and the intertrial interval (ITI) ensued; the ITI randomly ranged from 6 to 10 s. If the choice was incorrect, then food was not delivered, the houselight darkened, and a correction trial was scheduled. Correction trials were given until the correct response was made. No data were analyzed from correction trials.

*Intermediate tests (inter-tests).* Accuracy to each category was measured for each session. Our initial plan was to give a first testing session after each bird reached accuracy criteria of 55% correct for each category, then after they reached 60%, 65%, 70%, 75%, 80%, and 85% correct. However, some birds were fast learners; they skipped one or more lower criteria on their way to their highest criterion. Thus, we could not collect data for all of the different criteria for each of the birds in these intermediate tests (inter-tests); still, we could record the individual training trends, as we show in the *Results* section. Regardless of the specific criteria met, once a bird reached a given accuracy criterion, a testing session was scheduled for the next day.

Testing sessions began with eight warm-up training trials. The next 156 trials contained 120 training trials plus 36 randomly interspersed testing trials: four Prototype, eight Incongruent, four Only-D, eight Only-P, and 12 One-Absent-P trials. On training trials, only the correct response was reinforced; incorrect responses were followed by correction trials (differential reinforcement). On testing trials, any choice response was reinforced (non-differential reinforcement); food was given regardless of the pigeons’ choice responses, so that testing could proceed without explicitly teaching the birds the correct responses to the testing stimuli. No correction trials were given on testing trials.

After each inter-test, a bird would resume training until accuracy reached the next criterion. At least one training session had to be completed between inter-test sessions. Then, another testing session would be scheduled, followed again by training sessions. This process repeated until the bird reached 90% accuracy to both categories. Once this criterion was achieved, the bird began the final testing phase.

*Final testing phase.* The final phase consisted of 12 testing sessions, as we had done in Castro et al. (2020), so we could compare the pigeons’ current performance to those prior results. Also, having more data points allowed us to achieve a more stable output from the computational modeling.

Each of these testing sessions was the same as the inter-test sessions. No training sessions were scheduled between testing sessions. However, if at any point during these 12 sessions a bird’s accuracy in one training category dropped below 70%, then testing was paused, and the bird received additional training sessions until accuracy scores again reached the 90% criterion. Then, testing resumed from where the pigeon left off until all 12 sessions were completed.

### Data analysis

All data files are available in the OSF repository at https://osf.io/e35qv/?view_only=a9a9ad60883249b19fbc1859ae582e17

*Choice accuracy and peck tracking.* To analyze the birds’ performance, we measured: (1) their accuracy to categorize each stimulus exemplar and (2) the location of their pecks during the observing requirement. The birds could peck anywhere within the 10 × 10 cm stimulus area to complete the observing requirement before making their category choice response. We calculated the birds’ percentage of pecks within the 2.7 × 2.7 cm area occupied by each of the features over the total number of pecks to the entire 10 × 10 cm area occupied by the category exemplar on a daily basis. A peck was considered to be within the feature when the X and Y coordinates of the peck fell into the area occupied by a square tightly surrounding each of the features (see graphical representation in the Online Supplemental Material, OSM). All analyses were conducted using R, version 3.3.2 (R Development Core Team, 2016).

*Computational modeling.* To determine to what extent pigeons’ attention was selective and focused on a single feature or distributed across some or all of the features, choice responses to the testing items were analyzed using Nosofsky’s (1986, 1988, 1992) GCM.

A core assumption of GCM is that organisms represent categories by storing individual training exemplars in memory. Later classification of novel testing exemplars is based on similarity comparisons between the novel exemplars and the stored exemplars. Thus, according to GCM, in the case of two mutually exclusive categories, A and B, the probability that Stimulus S_*i*_ is classified in Category C_*A*_, that is, P(R_*A*_| S_*i*_), is given by the following equation:

$$P({R}_{A}|{S}_{i}) = \frac{{b}_{A} {\sum }_{a\in {C}_{A}}{sim}_{ia}}{{b}_{A} {\sum }_{a\in {C}_{A}}{sim}_{ia} +(1-{b}_{A} ){\sum }_{b\in {C}_{B}}{sim}_{ib}}$$

where *b*_*A*_ (0 ≤ *b*_*A*_ ≤ 1) is Category A response bias and *sim*_*ia*_ and *sim*_*ib*_ are similarities between a given exemplar *i* and exemplars belonging to Categories A and B, respectively. Similarity between items is an exponential decay function of psychological distance *d* derived from this equation:

$${sim}_{ia}= {e}^{{-cd}_{ia}}$$

where *c* (0 ≤ *c* ≤ ∞) is a sensitivity parameter reflecting overall discriminability in the psychological space, with larger values representing greater discriminability. Psychological distance *d* is calculated according to the following equation:

$${d}_{ia} = {\left[\sum_{m=1}^{M}{w}_{m }{|{x}_{im}- {x}_{am} |}^{r}\right]}^{{~}^{1}\!\left/ \!{~}_{r}\right.}$$

where *x*_*im*_ is the psychological value of exemplar *i* on dimension *m*. The value of the parameter *r* typically takes values of 1 or 2, for separable or integral dimensions, respectively (Shepard, 1991); given the characteristics of our stimuli, we used an *r* = 1, that results in the city-block metric as the distance between exemplars in multidimensional space. Most critically, *w*_*m*_ (0 ≤ *w*_*m*_ ≤ 1) is the attentional weight given to a dimension or feature *m*. These attentional weights are interpreted as reflecting the attention that is allocated to each dimension during categorization (Nosofsky, 1986; Viken, Treat, Nosofsky, McFall, & Palmeri, 2002). Attentional weights are of critical importance because they may change as a result of categorization training (Nosofsky, 1986); therefore, these weights reflect the amount of attention allocated to a particular feature at a given point in time. Because GCM assumes a limited-capacity system, the sum of all of the attentional weights equals 1; so, the more attention is paid to a particular feature, the less attention is paid to the other features.

As in Castro et al. (2020), we used GCM to estimate the attentional weights that best accounted for each pigeon’s responses on the categorization testing trials. To do so, we assumed that all of the exemplars presented during training were in the memory of the GCM. In order to get a close assessment of the attentional weights, we fixed the sensitivity parameter (*c* = 3, which is often estimated from data) and adopted a city-block metric (*r* = 1). By means of numerical optimization, we found the attentional weights that minimized the sum of squared error (SSE) between the GCM’s and a given pigeon’s responses to the stimuli presented during each testing phase, using all the trial data for that specific testing phase.

Once we obtained the vector of attentional weights for each pigeon, we summarized each pigeon’s attentional profile by calculating the entropy of their vector of attentional weights. Because we were interested in determining whether the pigeons focused on one feature or distributed their attention over some or all of them, we considered that *entropy*, a measure of variety or diversity provided by information theory (Shannon & Weaver, 1949), was a good candidate for this purpose. Entropy measures the amount of informational diversity by computing a weighted average of the amount of information that, in our case, each of the features in an exemplar provides to a given pigeon.

If a pigeon is allocating all its attention to a single feature, then this single feature carries all of the information (in this case, the vector of attentional weights for our five-feature stimulus would be: *w* = {1, 0, 0, 0, 0}); then, there is no informational diversity, so entropy is 0. If a pigeon is distributing its attention across multiple features, then several features carry some amount of information, and entropy is large. Entropy is maximal when a pigeon is using all of the features to the same extent, so that each of them carries an equal amount of information (e.g., *w* = {.2,.2,.2,.2,.2}). We used the following equation to calculate the entropy of the vectors of attentional weights for each pigeon:

$${H}_{w}= - \sum_{m}^{M}\mathrm{log}({w}_{m}){w}_{m}$$

where ${H}_{w}$ is the entropy of the vector of attentional weights *w*, and *w*_*m*_ is the attentional weight of each feature *m*. Once entropy for each pigeon was obtained, it was then normalized based on the maximum possible entropy given the length of the vector of weights (uniform distribution of five weights). Thus, normalized entropy was bound between 0 and 1, with 0 representing maximal selectivity (when the attentional weight for one of the features equals 1, whereas the remaining weights for all of the other features equals 0), and with 1 representing minimal selectivity or maximal distribution of attention (when all of the attentional weights are equal).

Computational modeling was conducted using the catlearn package for R developed by Andy Wills (https://www.andywills.info/catlearn/). We modified the code to adapt it to our specific stimuli and experimental design. In addition, we wrote the code to calculate the entropy of the attentional weights. Our scripts can be found on the OSF at https://osf.io/e35qv/?view_only=a9a9ad60883249b19fbc1859ae582e17

*Heat maps.* As an additional aid to visualize the spatial distribution of each pigeon’s pecks to the category stimuli, we created heat maps for the most critical exemplars. These heat maps, and the details about how they were created, can be found in the Online Supplemental Material (OSM).

## Results

All six pigeons reached the final target learning criterion (90% correct on each category), but at very different speeds. The fastest pigeons (56Y and 30Y) reached criterion in 5 days, whereas the slowest pigeon (17R) took 36 days. The other three pigeons, 56B, 37 W, and 48 W, took 17, 19, and 19 days, respectively. Our plan to test the pigeons after each 5% increment in accuracy proved to be impractical because, for example, some birds reached 70% accuracy for each category in just two sessions (pigeons 56Y and 30Y) or because they moved from one criterion to a much higher accuracy criterion immediately afterward (e.g., going from 75% directly to 85%) without going through more intermediate criteria. In addition, different pigeons exhibited very different performance patterns during training and in the final testing phase. Thus, we first report group performance in the final testing test, and then we analyze the data of each individual pigeon and their distinctive learning and attentional patterns throughout training.

### Final testing phase

**Fig. 2:**
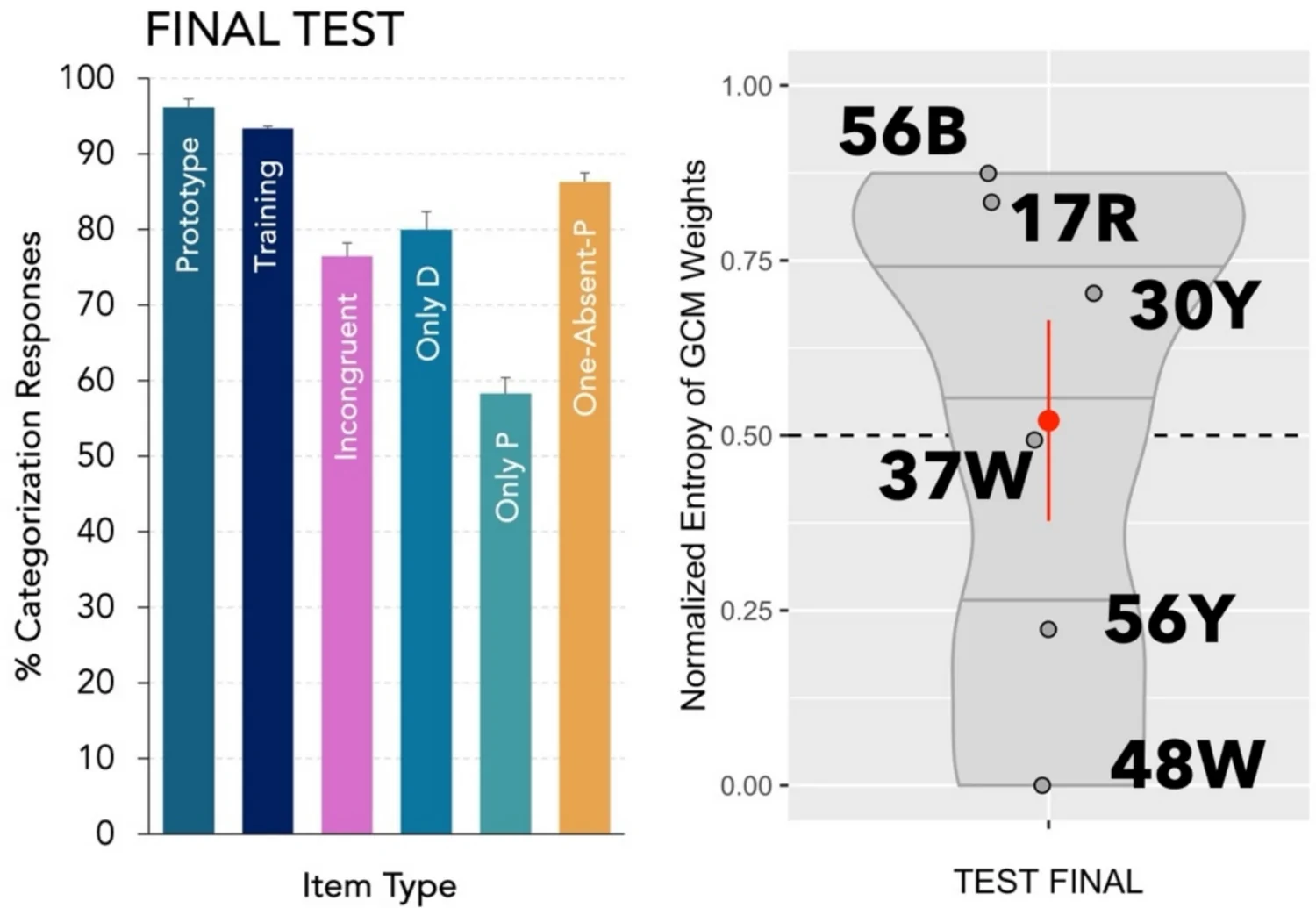
**Left:** Mean percentage of correct responses to the different types of items during the final testing phase. In the case of Incongruent items, responses based on the deterministic feature were considered to be correct. Error bars indicate the standard error of the means. **Right:** Normalized entropy of the Generalized Context Model (GCM)’s best-fitting attentional weights for each individual pigeon during the final testing phase. The violin plot depicts the density distributions of normalized entropy. A value of 0 represents maximal selectivity (when the attentional weight for one of the features equals 1 and the remaining weights for all other features equals 0), whereas a value of 1 represents minimal selectivity or maximal distribution of attention (when all weights are equal). Individual data points are horizontally jittered (randomly shifted left and right) to allow better visualization of each pigeon’s entropy score. The red point indicates the mean of the distribution. The dashed line, at .50, represents the middle value and is included as a reference

The pigeons’ mean categorization responses to training and testing trials during the final testing phase are shown in Fig. 2, left. Although, from the pigeons’ perspective, there were no correct or incorrect responses on testing trials, we considered correct those category responses that matched the category represented by the deterministic feature or the probabilistic features, as learned during training. The case of Incongruent trials posed a special problem, because the deterministic and probabilistic features were put into conflict; in this case, responses based on the deterministic feature were considered to be correct.

Accuracy was very high for Training (*M* = 93.4%), Prototype (*M* = 96.1%), and One-Absent-P trials (*M* = 86.3%). On Incongruent trials, in which the deterministic and probabilistic features were put into conflict, the deterministic feature exerted greater control than the probabilistic features, thereby leading to substantially above-chance performance (*M* = 76.4%). Consistent with those choices, when the deterministic feature alone was available on Only-D trials, pigeons’ accuracy was also high (*M* = 79.9%), although clearly lower than their accuracy on Training trials. Accuracy was slightly above chance for Only-P trials (*M* = 58.3%), when only the probabilistic features were available. Overall, pigeons seemed to rely more on the deterministic feature than on the probabilistic features to solve the task.

To confirm these observations, we conducted a logistic mixed-effects model analysis with trial type (Training, Prototype, Incongruent, Only-D, Only-P, One-Absent-P) as the fixed effect (treatment coded, with Training trials as the reference condition). A log-likelihood ratio test indicated that the best-fitting random-effects structure included a random intercept for bird. Accuracy on Training trials was very high, *M* = 93.4, 95% CI_*M*_ [92.9, 93.8]. It was slightly higher on Prototype trials, *M* = 96.1, 95% CI_*M*_ [93.9, 98.4], suggesting that having all the probabilistic features from the correct category helped pigeons improve their accuracy. However, this improvement was not statistically significant, *B* = 0.52, *SE* = 0.44, *Z* = 1.18, *p* =.60.

When the deterministic and probabilistic features were put into conflict on Incongruent trials, pigeons tended to respond according to the category of the deterministic feature, *M* = 76.4, 95% CI_*M*_ [72.9, 79.9]. Despite the relatively large overall decrement in performance compared to Training trials, the difference was not statistically significant, *B* = −1.01, *SE* = 0.49, *Z* = 2.04, *p* =.14. As we will see later, this lack of statistical difference was due to the strikingly different performance of the various birds.

On Only-D trials, when only the deterministic feature was available, pigeons’ accuracy was high, *M* = 79.9, 95% CI_*M*_ [75.2, 84.6], but significantly lower than on Training trials, *B* = −1.08, *SE* = 0.32, *Z* = −3.39, *p* =.003. On Only-P trials, when only the probabilistic features were available, pigeons’ accuracy dropped much more, *M* = 58.3, 95% CI_*M*_ [54.2, 62.3] and was significantly lower compared to Training trials, *B* = −2.46, *SE* = 0.36, *Z* = −6.80, *p* <.001. Still, pigeons’ accuracy was significantly higher than the 50% chance level, *t*(5) = 2.33, *p* =.03. It seems, therefore, that the pigeons could also use at least some of the probabilistic features to perform the task. When only one of the probabilistic features was removed on One-Absent-P trials, pigeons’ accuracy was very high, *M* = 86.3, 95% CI_*M*_ [83.9, 88.6], although there was a small, but significant, decrease in accuracy compared to Training trials, *B* = −0.66, *SE* = 0.23, *Z* = −2.88, *p* =.016.

Thus, when the deterministic and probabilistic features were put into conflict on Incongruent trials, pigeons, overall, tended to rely on the deterministic feature more than on the probabilistic features, most likely because they had learned the perfect predictive value of the deterministic feature. However, pigeons’ decrease in accuracy on Only-D and One-Absent-P trials, along with their higher than chance accuracy on Only-P trials, suggest that at least some of the probabilistic features were having a measurable impact on pigeons’ performance as well.

The entropies of the pigeons’ attentional profiles resulting from the GCM fits are shown in Fig. 2, right. Even when the pigeons, as a group, had evidenced strong reliance on the deterministic feature to solve the categorization task, their individual attentional profiles were quite diverse. One pigeon, 48 W, exhibited maximal selectivity (entropy = 0), that is, complete focus on the single deterministic feature. A second pigeon, 56Y, received a very low entropy score (entropy =.22), suggesting high, but not total, focus on the deterministic feature. The other four pigeons showed varied levels of distributed attention (entropy values ranged from .49 to .87) to the several features in the category exemplars. Given this diversity in the pigeons’ attentional profiles, we looked at their individual learning rates, peck tracking, performance in the inter-tests, and GCM fits for the inter-tests, to better understand the complexity of their behavior.

### Training and intermediate tests

**Fig. 3:**
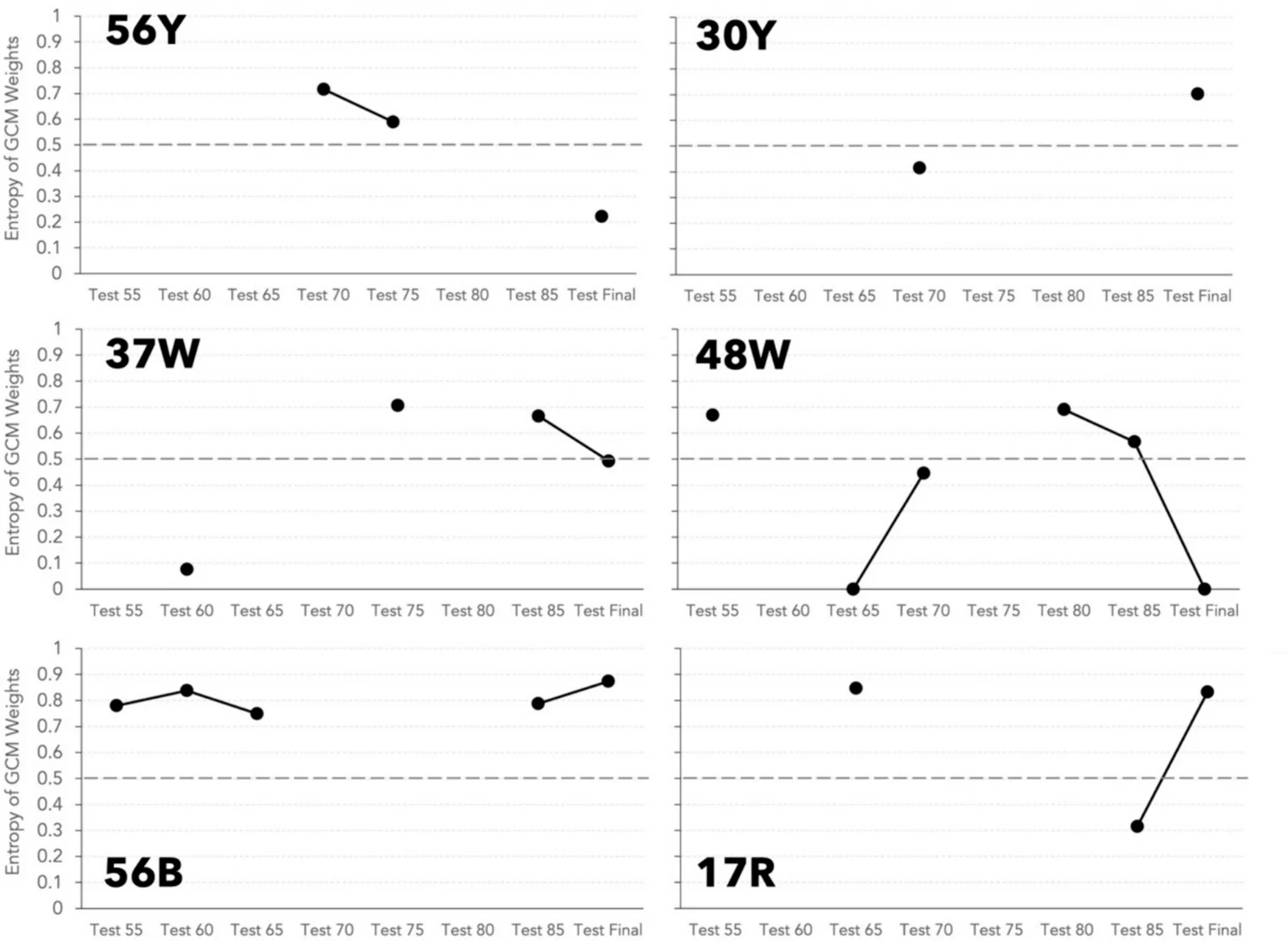
Normalized entropy of the Generalized Context Model (GCM)’s best-fitting attentional weights for each individual pigeon in the inter-tests and in the final test. The accuracy criterion met immediately before the test is indicated in the X axis. An accuracy criterion of 90% had to be met before starting the final testing phase. As explained in the text, pigeons’ accuracy progressed quite fast, so that they could not be tested after each of the possible intermediate accuracy criteria. Thus, a data point for a given pigeon will be depicted when a given criterion was met. For example, 56B met the 55% criterion and was tested, then the 60% criterion in next session and was tested, and the 65% criterion in a later session; after the inter-test following the 65% criterion, 56B met the 85% criterion right away, so no entropy values are available for him for Test 70, Test 75, and Test 80. A value of 0 represents maximal selectivity (when the attentional weight for one of the features equals 1 and the remaining weights for all other features equal 0), whereas a value of 1 represents minimal selectivity or maximal distribution of attention (when all weights are equal). The dashed line, at .50, represents the middle value and is included as a reference

To analyze the birds’ performance during training, we measured: (1) their accuracy to categorize each exemplar and (2) the location of their pecks during the presentation of each category exemplar prior to making the category choice response. The birds could peck anywhere within the area occupied by the category exemplar to complete the observing requirement before making their category choice response. We calculated the birds’ daily percentage of pecks within the area occupied by each of the features over the total number of pecks to the area occupied by the category exemplar. A large percentage of pecks at the deterministic feature (D Pecks) would be consistent with the pigeons focusing their attention on the deterministic feature. In addition, we used GCM to estimate the attentional weights just after the birds reached intermediate accuracy levels. Figure 3 depicts the entropies of each pigeon’s attentional profile derived from the GCM fits in the inter-tests. We will next examine how these changes in each of the pigeons’ attentional weights relate to their overall performance.

### Pigeons 56Y and 30Y

**Fig. 4:**
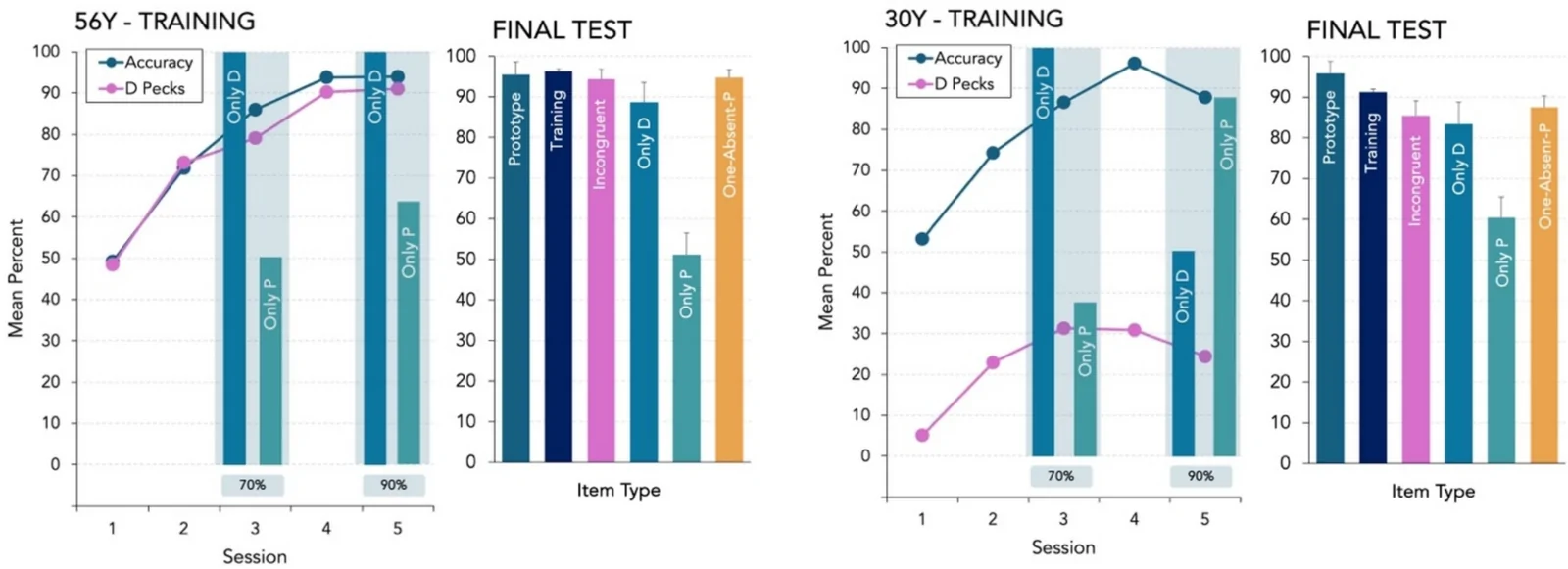
Training and final testing performance for 56Y (**left**) and 30Y (**right**). Mean accuracy and percentage of pecks at the deterministic feature (D Pecks) per session are shown. Inter-test sessions are marked by a blue shade; in these inter-test sessions, the percentage below refers to the criterion met the prior day. The inter-test session after 90% criterion is the first testing session of the final test (12 sessions). For the sake of clarity, we just include performance to Only-D and Only-P trials (the most informative) for the inter-tests (see text for further information). Error bars indicate the standard error of the means

As shown in Fig. 4 (left), Pigeon 56Y learned the task very quickly; her accuracy was around 50% in Session 1, and it surpassed 70% per category in Session 2. In these two sessions, as her accuracy increased, her D pecks increased as well (*M* = 73% in Session 2). Moreover, on Session 4, her accuracy reached 93% and almost all her pecks were directed to the deterministic feature (*M* = 90%). Heat maps in Fig. S2 in the OSM support these observations: during the first and second days of training, 56Y’s pecks were distributed between the deterministic and one of the probabilistic features, but they concentrated solely on the deterministic feature on Session 4. So, we could conclude that strong tracking of the deterministic feature allowed this bird to master the task in a very short period of time.

Pigeon 56Y was given her first inter-test in Session 3. Because Only-D and Only-P trials proved to be the most revealing, we will just report accuracy on those trials for the inter-tests (performance on all of the trials for each pigeon is available on the OSF at https://osf.io/e35qv/?view_only=a9a9ad60883249b19fbc1859ae582e17). On this first inter-test, 56Y’s accuracy was 100% on Only-D trials, when just the deterministic feature was present, and exactly at 50% chance on Only-P trials, when the deterministic feature was absent. Still, 56Y’s attentional profile revealed that her attention was actually quite distributed at this point (entropy =.72; see Test 70 in Fig. 3). It seems that, despite her strong tracking of the deterministic feature, she noticed the new testing stimuli, which diverted her attention (even when she was still pecking at the deterministic feature). After reaching the 90% accuracy criterion in Session 4, her testing accuracy on Session 5 was still consistent with her high tracking of the deterministic feature: 100% on Only-D trials, but only 63% on Only-P trials.

Over the 12 days in the final testing phase, 56Y’s pattern of responses, as before, showed a strong reliance on the deterministic feature, with high accuracy on Incongruent (M = 94%) and Only-D (M = 89%) trials and chance performance on Only-P trials (M = 51%). Her entropy score during the final test indicates a strong attentional focus (entropy =.22; see Fig. 3, Final Test). Thus, 56Y’s attention seemed to be diverted by other features the first time that novel testing stimuli were presented, but she returned her focus to the deterministic feature subsequently (see Fig. S2 in OSM as well).

Pigeon 30Y also learned the task very quickly (Fig. 4, right). His accuracy was 53% in Session 1, and it surpassed 70% per category in Session 2. Although his accuracy greatly increased over the chance level in these two sessions, his D pecks increased only slightly from 5% in Session 1 to 23% in Session 2. As the heat maps in Fig. S2 (OSM) show, 30Y was distributing his pecks among several features of the category exemplar, both at the beginning and at the end of training.

On the first inter-test, 30Y’s accuracy was 100% on Only-D trials, when just the deterministic feature was present, but below chance (*M* =37.5%) on Only-P trials, when the deterministic feature was absent. So, 30Y’s choice accuracy suggests that he discerned the predictive value of the deterministic feature but was not tracking it. The attentional profile computed from the first inter-test disclosed that his attention was fairly distributed (entropy =.42; see Test 70 in Fig. 3). His training accuracy reached 96% on the next day, so he was tested again in Session 5 (first testing session of the final testing phase). At this point, 30Y’s peck tracking of the deterministic feature had risen to 30% in Session 4, but fell slightly to 25% in Session 5. Choice accuracy to testing stimuli in Session 5 fit his peck tracking scores: at 50% chance on Only-D trials and 88% on Only-P trials. It seems that 30Y had learned to distribute his attention over various features of the category exemplars.

Performance in the final testing phase suggests that 30Y could use the deterministic feature, as shown by high accuracy on Incongruent (*M* = 85%) and Only-D (*M* = 83%) trials, and relatively low accuracy on Only-P trials (*M* = 60%). Still, 30Y seemed to be using some of the other features as well (only accuracy for Prototype and Training stimuli was higher than 90%), as the heat maps in Fig. S2 (OSM) reveal. His entropy score during the final test supports these observations: an entropy score of .70 indicates highly distributed attention for 30Y (see Fig. 3, Final Test).

### Pigeons 37 W and 48W

**Fig. 5:**
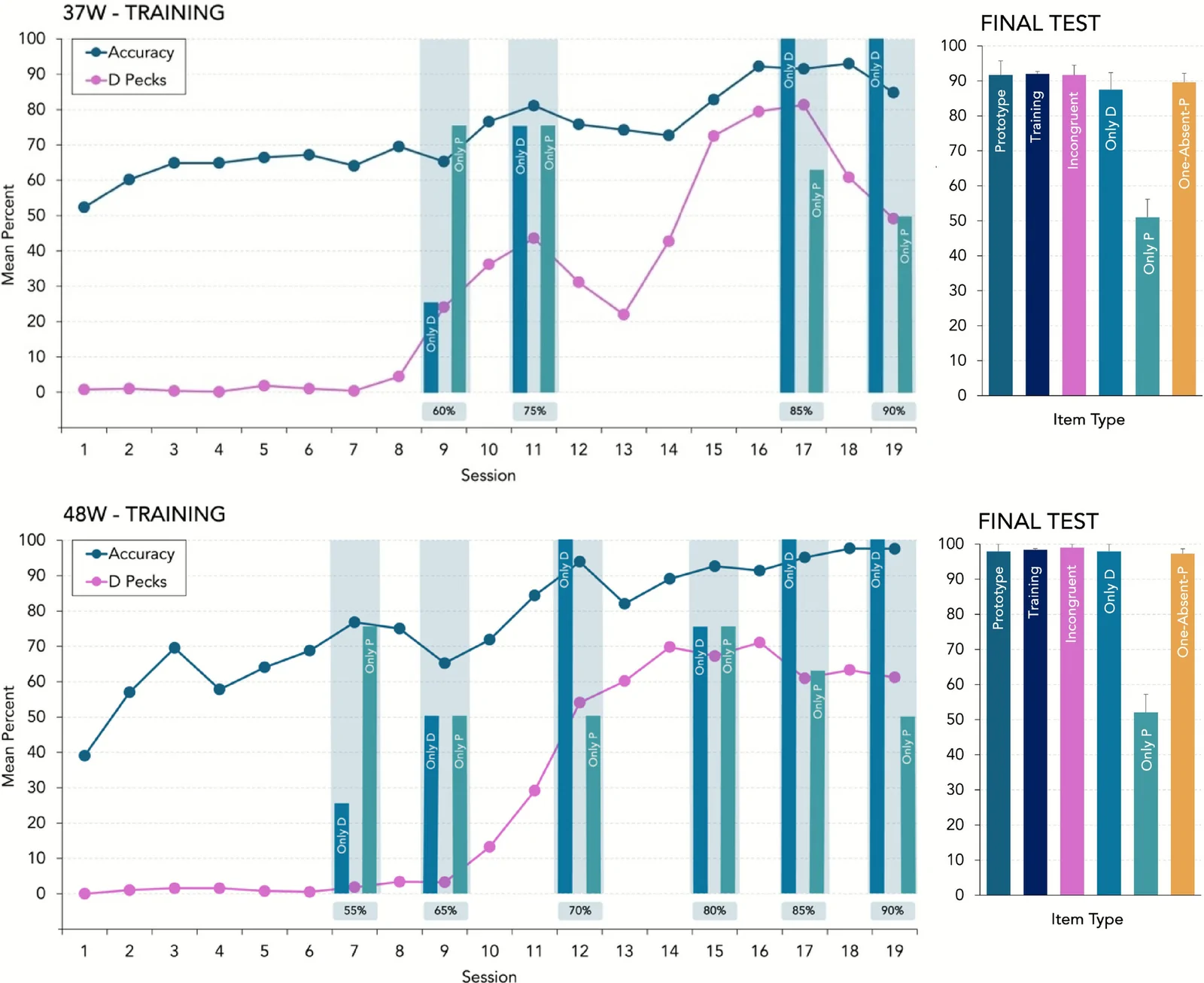
Training and final testing performance for 37 W (**top**) and 48 W (**bottom**). Mean accuracy and percentage of pecks at the deterministic feature (D Pecks) per session are shown. Inter-test sessions are marked by a blue shade; in these inter-test sessions, the percentage below refers to the criterion met the prior day. The inter-test session after 90% criterion is the first testing session of the final test (12 sessions). For the sake of clarity, we just include performance to Only-D and Only-P items (the most informative) for the inter-tests (see text for further information). Error bars indicate the standard error of the means

Pigeon 37 W met his first criterion, 60%, after 8 days of training. Pigeon 48 W took 6 days to reach the first learning criterion, 55%, before the first inter-test; after that first test, she met the next criterion, 65%, on the next session, so she was tested again the following day. These two birds never pecked the deterministic feature on those first training days; but, after those initial tests, both started tracking the deterministic feature and both were pecking it a high percentage of the time in the last training sessions (see Fig. 5, and heat maps in Fig. S3 in the OSM). Correspondingly, on their first inter-test, both birds’ accuracy on Only-D items was very low, but increased up to 100% on the last inter-tests. On the other hand, when only the probabilistic features were present, accuracy was around 70% for both birds on the first inter-test, but eventually decreased to 50% chance level, suggesting that the initial reliance on the probabilistic features faded when the birds learned the value of the deterministic feature. Interestingly, it seems that presentation of trials with only the deterministic feature and/or trials with only the probabilistic features were crucial for these two birds to start focusing on the deterministic feature.

High accuracy to Incongruent and Only-D trials, and chance performance when the deterministic feature was absent on Only-P trials indicates reliance on the deterministic feature in the 12 days of the final testing phase for both birds. Although 37W’s peck tracking had decreased a little at the very end of training, his peck tracking was fairly robust during the final test (*M* = 61%). Pigeon 48 W continued her upward trend and her peck tracking averaged 85% during the final test. Indeed, as can be seen in Fig. 3, 48W’s entropy scores show maximal selectivity on the final test (see also heat maps in Fig. S3, OSM). On the other hand, 37 W shows more distributed attention, although not as high as other birds. We should note that these two birds had an entropy score of 0 in two of the initial inter-tests (37W in Test 60, and 48 W in Test 65), indicating a complete focus on one of the features. However, the focus was not on the deterministic feature; both 37 W and 48 W were tracking the probabilistic feature on the bottom right at that point (see heat maps from the beginning of training in Fig. S3, OSM).

### Pigeons 56B and 17R

**Fig. 6:**
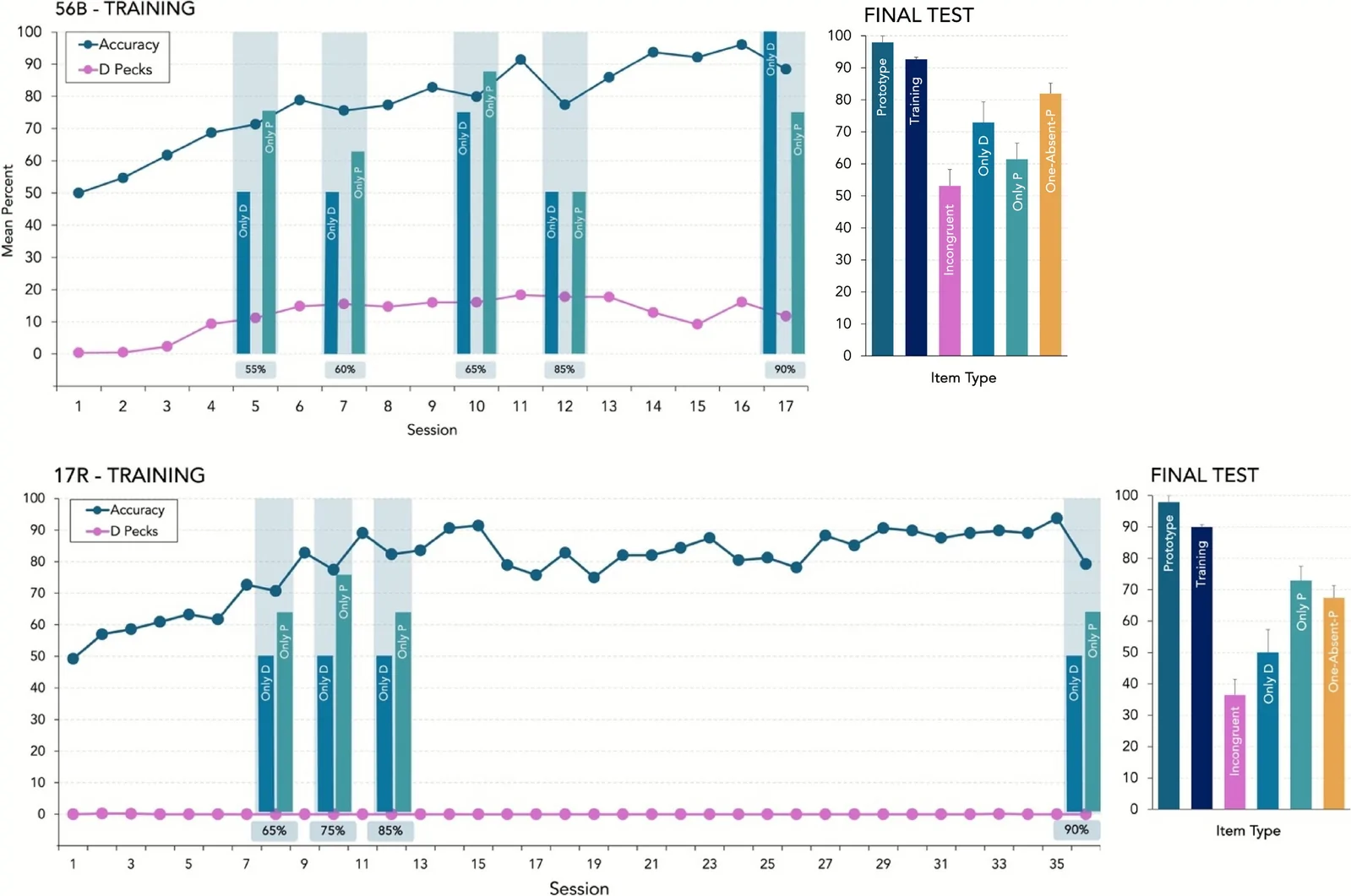
Training and final testing performance for 56B (**top**) and 17R (**bottom**). Mean accuracy and percentage of pecks at the deterministic feature (D Pecks) per session are shown. Inter-test sessions are marked by a blue shade; in these inter-test sessions, the percentage below refers to the criterion met the prior day. The inter-test session after 90% criterion is the first testing session of the final test (12 sessions). For the sake of clarity, we just include performance to Only-D and Only-P trials (the most informative) for the inter-tests (see text for further information). Error bars indicate the standard error of the means

Pigeon 56B took 4 days to reach the first learning criterion, 55%, before his first inter-test. Pigeon 17R met her first criterion, 65%, after 7 days of training. At that point, these birds were not tracking the deterministic feature and, in their first inter-test, they responded at chance when only the deterministic feature was presented (see Fig. 6). These pigeons’ accuracy on Only-P trials, when exemplars containing only the probabilistic features were presented, was very close to their training performance, indicating that they were relying on the overall similarity of the exemplars.

As was the case for birds 37 W and 48 W, after the first inter-test, pigeon 56B started pecking at the deterministic feature more often, although never more than 20% of his pecks were directed to this feature (his pecks were distributed among the deterministic and the probabilistic features on the right side of the category exemplar, as shown in the heat map in Fig. S4, OSM). Pigeon 17R never pecked the deterministic feature. After that first test, these birds met the next criteria, 85%, relatively quickly. It took 56B 4 more days to reach the 90% criterion. At this point, 56B, who was tracking the deterministic feature to some extent, scored 100% on Only-D trials, showing that he had learned the value of the deterministic feature. On the other hand, 17R took 23 more days to reach the 90% criterion. This bird never pecked at the deterministic feature and, on the last inter-test, it remained at chance level on Only-D trials. In the final testing phase, 56B’s mean accuracy on Only-D trials was 73%, indicating that he was relying as well on the probabilistic features (on Training trials, in which the deterministic and probabilistic features were presented, his accuracy was 93%). Indeed, 56B’s near-chance responding to Incongruent trials (*M* = 53%) reflects the conflict between the deterministic (focusing on one category) and the probabilistic (focusing on other category) features. On the other hand, 17R’s below-chance responding on Incongruent trials (*M* = 36%) indicates that the probabilistic features exerted greater control over her category choices than the deterministic feature (remember that a high score on Incongruent trials reflects reliance on the deterministic feature); still, because her training accuracy (*M* = 90%) was higher than her accuracy on Only-P trials, we conclude that she was probably relying on some combination of the deterministic and some probabilistic features (her pecks were directed mostly at the bottom features of the category exemplar, as shown in the heat maps in Fig. S4, OSM). The low reliance on the deterministic feature of these two pigeons is consistent with their high entropy scores (see Fig. 3, Final Test).

## Discussion

All of our pigeons mastered a categorization task that could be solved by focusing attention on a single deterministic feature or by distributing attention across multiple probabilistic features. After reaching 90% accuracy levels for both categories, pigeons’ overall performance indicated that they relied predominately on the deterministic feature, the most predictive feature – their accuracy declined to 80% when only the deterministic feature was present, and they preferred the deterministic over the probabilistic features when the two types of testing stimuli were put into conflict. However, the pigeons also attended to, or were distracted by, other, albeit less diagnostic information – their accuracy was not as high when only the deterministic feature was present, compared to their accuracy with the category prototype which contained the deterministic feature and all of the probabilistic features from the correct category. Thus, pigeons tended to divert their attention from the most predictive feature, even when they clearly perceived its value.

These results also further support the validity of peck tracking as a method to measure attention in birds. In humans, eye tracking is widely regarded as a valid measure of attentional allocation. For example, in a categorization task in which – similar to our procedure – the features comprising the stimuli were spatially separated, Rehder and Hoffman (2005a) found that, as learning progressed, participants’ gaze direction gradually shifted toward the features relevant for correct classification (see also Blair et al., 2009; Uengoer et al., 2018), a pattern that mirrors our peck-tracking findings in pigeons (Castro & Wasserman, 2014, 2016, 2017; Castro et al., 2021; Sheridan et al., 2019). In addition, Rehder and Hoffman reported that eye movements toward relevant stimulus elements tended to follow, rather than precede, improvements in categorization accuracy. Although we did not examine this relationship in the present experiment, Castro and Wasserman (2017) analyzed their peck-tracking data using computational modeling and similarly found that increases in pecking at relevant features followed gains in categorization accuracy. Taken together, these findings suggest that peck tracking can serve as a reliable measure of attention in pigeons (see also Dittrich et al., 2010), much as eye tracking is considered a reliable measure of attention in humans (see Rehder & Hoffman, 2005b, for a review).

Interestingly, the amount of attentional allocation to the deterministic feature was highly varied across pigeons. Two of the six pigeons (56Y and 48 W) showed a strong focus on the deterministic feature. Pigeon 56Y concentrated on the deterministic feature very early in training and, consequently, she learned the category discrimination very rapidly. Pigeon 48 W took longer to learn, but this was due to her initial focus on another *single* feature; once she shifted her pecks to the deterministic feature, her accuracy greatly increased and she ended up being the most selective pigeon, pecking exclusively at the deterministic feature. Pigeon 37 W was also quite focused on the deterministic feature, although a proportion of his pecks was also directed to one of the probabilistic features. Pigeons 30Y and 56B showed very distributed pecking patterns, allocating their pecks among three of the features, including the deterministic feature. Finally, Pigeon 17R never pecked the deterministic feature; she distributed her pecks between two of the probabilistic features.

Individual differences are recognized and presumed to be of considerable importance in human research (e.g., Kanai & Rees, 2011; Underwood, 1975), but often ignored in animal research, under the assumption that animals’ circumstances are less variable and that their behavior tends to be more stereotypical, therefore ignoring their genetics, breeding, prior experiences, and memories. Thus, variability across individuals is typically considered a nuisance that impairs the researcher’s capacity to detect the effect of an independent variable. After all, we seek to discover explanatory principles at a general level rather than at the individual level. However, these should not be mutually exclusive goals. Rather than assuming common performance patterns in all of the individuals of a given population, differences among their members should be considered as well (e.g., Barsalou, 2020). Our analyses show that trying to simplify and unduly summarize a given set of data may lead to less accurate information and to an incomplete understanding of the animals’ behavior.

The combination of an individual differences approach along with the pursuit of general cognitive principles has lately been emphasized (e.g., Kirchhoff & Buckner, 2006; Vogel & Awh, 2008; Wilmer & Nakayama, 2007). Our pigeons displayed different patterns of performance, but we can extract important general conclusions from their behavior as well. All pigeons started distributing their attention among several features of the stimuli; focusing on just one feature (for those who did) emerged as training progressed. Critically, our results showed that pigeons’ peck tracking precisely aligns with their category choices. For example, the more focused the pigeons’ overall pecks were on the deterministic feature, the more likely they were to be able to correctly categorize the stimuli that contained only the deterministic feature and the more likely they were to rely on the deterministic feature when the deterministic and probabilistic features were put into conflict. This pattern of behavior validates the value of peck tracking as a proxy for pigeons’ attention – in other words, for the stimulus information that controls the pigeons’ categorization behavior (see also Castro & Wasserman, 2014, 2016, 2017; Castro et al., 2021; Dittrich et al., 2010; Sheridan et al., 2019).

In addition, our data encourage us to make predictions about future findings. Specifically, pigeons’ particular tracking patterns should allow us to predict performance in subsequent tasks. For example, those pigeons who focused exclusively on the deterministic feature should have more difficulty learning a later reversal task in which the weakly predictive features become the highly predictive features, and vice versa. Also, those pigeons who distributed their attention among several weakly predictive features should be more likely to detect and to learn the value of a highly predictive feature that is added later.

Moreover, data on individual variability could be used to refine any given theory. Let us examine an example. Pigeons do not have a prefrontal cortex (PFC), and their pallium is nucleated and lacks the distinctive laminar organization observed in the mammalian cortex (Jarvis et al., 2005). Nevertheless, their forebrains are also modular, consisting of small networks with a connective core of hub nodes that includes structures (the nidopallium caudolaterale, NCL) similar to the mammalian PFC. These hub nodes are centrally located and richly connected (Shanahan, Bingman, Shimizu, Wild, & Güntürkün, 2013). Thus, several studies have found that the pigeons’ NCL is involved in many of the characteristic prefrontal functions: attention and executive control (Rose & Colombo, 2005), and specifically visual categorization (e.g., Güntürkün et al., 2021; Pusch et al., 2023). Taking these facts into account, we could examine pigeons’ individual neural activity and see whether a more selective focus on the deterministic feature is associated with greater activation in the NCL. Individual differences at the behavioral level should correlate with individual differences in neural activity, thereby strengthening our understanding of how different brain systems contribute to cognition.

We should also mention that, despite the lower predictive value of the probabilistic features, these features still conveyed some (albeit less reliable) information about category membership; such informativeness could have prevented the pigeons from totally ignoring them. Although this may be the case for some of the pigeons, we did observe that half of them (56Y, 37 W, and 48 W) did learn to ignore the probabilistic features by the end of training; these birds’ accuracy scores on Prototype, Training, and Incongruent trials did not differ, despite these stimuli having none, one, or three features, respectively, belonging to the opposite category (see Figs. 4 and 5 for their individual accuracy scores in the Final Test). Thus, pigeons may be capable of both focusing on the most relevant task information and simultaneously filtering out less relevant and totally irrelevant information (see, e.g., Corbetta & Shulman, 2002; Gulbinaite et al., 2014; Lennert & Martinez-Trujillo, 2011, for a distinction between focusing and filtering in selective attention).

Another point to consider is that the extent to which pigeons, or any other organism, focus on specific information or distribute their attention more broadly among multiple informational sources likely depends on the specific structure of the task stimuli (Garner, 1978). In our case, the stimuli comprised five discrete features that were spatially separated. This particular structure is likely to promote focus on one single feature rather than distributed attention. In contrast, discrete features that are closely located may instead promote a more comprehensive attentional scope. When dimensions rather than discrete features are used, and when these dimensions spatially overlap (as in the case of Gabor patch stimuli; e.g., Smith et al., 2012), differential attention to the most relevant dimension may depend on the salience of that dimension (O’Donoghue, Broschard, & Wasserman, 2020). So, attentional strategies in pigeons are probably influenced by the spatial and structural properties of the task stimuli. Understanding these relationships between stimulus structure and attentional allocation patterns should be thoroughly examined in future experimental designs.

Finally, Lea and his colleagues (Lea & Wills, 2008; Lea et al., 2009, Nicholls et al., 2011, Wills et al., 2009) have argued that, when given complex discrimination tasks, pigeons have a strong tendency to come under the control of single features. However, we have seen here that this is not a general tendency. Half of our pigeons showed preferential, even exclusive, use of the deterministic features; but the other half of our pigeons were more inclined to use several of the available features. Given the unique structure of the present task, both strategies could eventually lead to high accuracy scores. Further research where different strategies can lead to more disparate accuracy levels could help advance our understanding of the interaction between highly individual dispositions (e.g., to distribute or to focus attention) and more general patterns of behavior.

## Supplementary Information

Below is the link to the electronic supplementary material.

## Data Availability

The data and materials for the experiment are available via the Open Science Framework repository at https://osf.io/e35qv/?view_only=a9a9ad60883249b19fbc1859ae582e17 The experiment was not preregistered.

## Footnotes

1 In an early pilot peck-tracking experiment, we observed that, when the features of the category exemplars were close to one another, tracking of the relevant features was weak and unreliable; so, we placed the individual features farther apart in subsequent experiments. In Castro et al. (2020), because we were interested in seeing whether pigeons would attend to one or all of the features, and because it could have been difficult for pigeons to perceive all of the features if they were placed far from one another, we created stimuli in which the features were closer together; that is why we did not record the location of their pecks.

## Code availability

The R files used to analyze the data are available via the Open Science Framework repository at https://osf.io/e35qv/?view_only=a9a9ad60883249b19fbc1859ae582e17

## References

[CR1] Aust, U., & Braunöder, E. (2015). Transfer between local and global processing levels by pigeons (*Columba livia*) and humans (*Homo sapiens*) in exemplar- and rule-based categorization tasks. *Journal of Comparative Psychology,**129*(1), 1–16. 10.1037/a0037691 25150965

[CR2] Aust, U., & Huber, L. (2003). Elemental versus configural perception in a people-present/people-absent discrimination task by pigeons. *Learning & Behavior,**31*(3), 213–224. 10.3758/BF03195984 14577546

[CR3] Barsalou, L. W. (2020). Challenges and opportunities for grounding cognition. *Journal of Cognition,**3*(1), Article 31. 10.5334/joc.116 33043241 PMC7528688

[CR4] Blair, M. R., Watson, M. R., Walshe, R. C., & Maj, F. (2009). Extremely selective attention: Eye-tracking studies of the dynamic allocation of attention to stimulus features in categorization. *Journal of Experimental Psychology: Learning, Memory, and Cognition,**35*, 1196–1206. 10.1037/a0016272 19686015

[CR5] Brainard, D. H. (1997). The psychophysics toolbox. *Spatial Vision,**10*, 433–436. 9176952

[CR6] Castro, L., & Wasserman, E. A. (2014). Pigeons’ tracking of relevant attributes in categorization learning. *Journal of Experimental Psychology: Animal Learning and Cognition,**40*, 195–211. 10.1037/xan0000022 24893218

[CR7] Castro, L., & Wasserman, E. A. (2016). Attentional shifts in categorization learning: Perseveration but not learned irrelevance. *Behavioural Processes,**123*, 63–73. 10.1016/j.beproc.2015.11.001 26548717

[CR8] Castro, L., & Wasserman, E. A. (2017). Feature predictiveness and selective attention in pigeons’ categorization learning. *Journal of Experimental Psychology: Animal Learning and Cognition,**43*, 231–242. 10.1037/xan0000146 29120213 PMC5684886

[CR9] Castro, L., Savic, O., Navarro, V., Wasserman, E. A., & Sloutsky, V. M. (2020). Selective and distributed attention in human and pigeon category learning. *Cognition*. 10.1016/j.cognition.2020.104350 32634739 PMC7494638

[CR10] Castro, L., Remund Wiger, E., & Wasserman, E. (2021). Focusing and shifting attention in pigeon category learning. *Journal of Experimental Psychology. Animal Learning and Cognition,**47*(3), 371–383. 10.1037/xan0000302 34618535 PMC8514057

[CR11] Cerella, J. (1977). Absence of perspective processing in the pigeon. *Pattern Recognition,**9*, 65–68. 10.1016/0031-3203(77)90016-4

[CR12] Clark, W., & Colombo, M. (2022). Seeing the forest for the trees, and the ground below my beak: Global and local processing in the pigeon’s visual system. *Frontiers in Psychology,**13*, Article 888528. 10.3389/fpsyg.2022.888528 35756294 PMC9218864

[CR13] Cook, R. G. (2001). Hierarchical stimulus processing by pigeons. In R. G. Cook (Ed.), *Avian visual cognition. Available *http://www.pigeon.psy.tufts.edu/avc/cook/default.htm

[CR14] Cook, R. G., Wright, A. A., & Kendrick, D. F. (2019). Visual categorization by pigeons. *Behavioral approaches to pattern recognition and concept formation* (pp. 187–214). Psychology Press.

[CR15] Corbetta, M., & Shulman, G. L. (2002). Control of goal-directed and stimulus-driven attention in the brain. *Nature Reviews Neuroscience,**3*, 201–215. 10.1038/nrn755 11994752

[CR16] Dittrich, L., Rose, J., Buschmann, J.-U., Bourdonnais, M., & Güntürkün, O. (2010). Peck tracking: A method for localizing critical features within complex pictures for pigeons. *Animal Cognition,**13*, 133–143. 19557444 10.1007/s10071-009-0252-x

[CR17] Emmerton, J., & Delius, J. D. (1980). Wavelength discrimination in the ’visible’ and ultraviolet spectrum by pigeons. *Journal of Comparative Physiology. A, Neuroethology, Sensory, Neural, and Behavioral Physiology,**141*, 47–52. 10.1007/bf00611877

[CR18] Fremouw, T., Herbranson, W. T., & Shimp, C. P. (1998). Priming of attention to local or global levels of visual analysis. *Journal Of Experimental Psychology: Animal Behavior Processes,**24*(3), 278–290. 10.1037/0097-7403.24.3.278 9679305

[CR19] Fremouw, T., Herbranson, W. T., & Shimp, C. P. (2002). Dynamic shifts of pigeon local/global attention. *Animal Cognition,**5*(4), 233–243. 12461601 10.1007/s10071-002-0152-9

[CR20] Garner, W. R. (1978). Aspects of a stimulus: Features, dimensions, and configurations. *Cognition and categorization* (pp. 99–133). Routledge.

[CR21] George, D. N., & Pearce, J. M. (2012). A configural theory of attention and associative learning. *Learning & Behavior,**40*, 241–254. 22926999 10.3758/s13420-012-0078-2

[CR22] Gibson, B. M., Wasserman, E. A., Frei, L., & Miller, K. (2004). Recent advances in operant conditioning technology: A versatile and affordable computerized touch screen system. *Behavior Research Methods,**36*(2), 355–362. 10.3758/bf03195582 15354702

[CR23] Goto, K., Wills, J., & Lea, S. E. G. (2004). Global-feature classification can be acquired more rapidly than local-feature classification in both humans and pigeons. *Animal Cognition,**7*, 109–113. 10.1007/s10071-003-0193-8 15069610

[CR24] Gulbinaite, R., Johnson, A., de Jong, R., Morey, C. C., & van Rijn, H. (2014). Dissociable mechanisms underlying individual differences in visual working memory capacity. *NeuroImage,**99*, 197–206. 10.1016/j.neuroimage.2014.05.060 24878830

[CR25] Güntürkün, O. (2005). The avian ‘prefrontal cortex’ and cognition. *Current Opinion in Neurobiology,**15*, 686–693. 16263260 10.1016/j.conb.2005.10.003

[CR26] Güntürkün, O. (2012). The convergent evolution of neural substrates for cognition. *Psychological Research,**76*, 212–219. 10.1007/s00426-011-0377-9 21881941

[CR27] Güntürkün, O., von Eugen, K., Packheiser, J., & Pusch, R. (2021). Avian pallial circuits and cognition: A comparison to mammals. *Current Opinion in Neurobiology,**71*, 29–36. 10.1016/j.conb.2021.08.007 34562800

[CR28] Huber, L., & Aust, U. (2012). A modified feature theory as an account of pigeon visual categorization. In T. R. Zentall & E. A. Wasserman (Eds.), *The Oxford handbook of comparative cognition* (pp. 497–512). Oxford University Press.

[CR29] Jarvis, E. D., Güntürkün, O., Bruce, L., Csillag, A., Karten, H., Kuenzel, W., Medina, Loreta, Paxinos, George, Perkel, David J.., Shimizu, Toru, Striedter, Georg, Wild, J. Martin.., Ball, Gregory F.., Dugas-Ford, Jennifer, Durand, Sarah E.., Hough, Gerald E.., Husband, Scott, Kubikova, Lubica, Lee, Diane W.., & Butler, A. B. (2005). Avian brains and a new understanding of vertebrate brain evolution. *Nature Reviews Neuroscience,**6*, 151–159. 10.1038/nrn1606 15685220 PMC2507884

[CR30] Kanai, R., & Rees, G. (2011). The structural basis of inter-individual differences in human behaviour and cognition. *Nature Reviews Neuroscience,**12*, 231–242. 10.1038/nrn3000 21407245

[CR31] Kirchhoff, B. A., & Buckner, R. L. (2006). Functional-anatomic correlates of individual differences in memory. *Neuron,**51*(2), 263–274. 16846860 10.1016/j.neuron.2006.06.006

[CR32] Kruschke, J. K. (1992). Alcove: An exemplar-based connectionist model of category learning. *Psychological Review,**99*, 22–44. 1546117 10.1037/0033-295x.99.1.22

[CR33] Lazareva, O. F., & Wasserman, E. A. (2010). Category learning and concept learning in birds. In D. Mareschal, P. C. Quinn, & S. E. G. Lea (Eds.), *The making of human concepts. *Oxford University Press.

[CR34] Lea, S. E. G., & Wills, A. J. (2008). Use of multiple dimensions in learned discriminations. *Comparative Cognition and Behavior Reviews,**3*, 115–133.

[CR35] Lea, S. E. G., Wills, A. J., Leaver, L. A., Ryan, C. M. E., Bryant, C. M. L., & Millar, L. (2009). A comparative analysis of the categorization of multidimensional stimuli: II. Strategic information search in humans (*Homo sapiens*) but not in pigeons (*Columba livia*). *Journal of Comparative Psychology,**123*, 406–420. 10.1037/a0016851 19929109

[CR36] Lennert, T., & Martinez-Trujillo, J. (2011). Strength of response suppression to distracter stimuli determines attentional-filtering performance in primate prefrontal neurons. *Neuron,**70*, 141–152. 10.1016/j.neuron.2011.04.011 21482363

[CR37] Mackintosh, N. J. (1965). Selective attention in animal discrimination learning. *Psychological Bulletin,**64*, 124–150. 14320077 10.1037/h0022347

[CR38] Mackintosh, N. J. (1975). A theory of attention: Variations in the associability of stimuli with reinforcement. *Psychological Review,**82*, 276–298.

[CR39] Nicholls, E., Ryan, C. M. E., Bryant, C. M. L., & Lea, S. E. G. (2011). Labeling and family resemblance in the discrimination of polymorphous categories by pigeons. *Animal Cognition,**14*, 21–34. 20652343 10.1007/s10071-010-0339-4

[CR40] Nosofsky, R. M. (1986). Attention, similarity, and the identification–categorization relationship. *Journal of Experimental Psychology: General,**115*, 39–57. 10.1037/0096-3445.115.1.39 2937873

[CR41] Nosofsky, R. M. (1988). Exemplar-based accounts of relations between classification, recognition, and typicality. *Journal of Experimental Psychology. Learning, Memory, and Cognition,**14*, 700–708.

[CR42] Nosofsky, R. M. (1992). Similarity scaling and cognitive process models. *Annual Review of Psychology,**43*, 25–53.

[CR43] O’Donoghue, E. M., Broschard, M. B., & Wasserman, E. A. (2020). Pigeons exhibit flexibility but not rule formation in dimensional learning, stimulus generalization, and task switching. *Journal of Experimental Psychology. Animal Learning and Cognition,**46*, 107–123. 31916780 10.1037/xan0000234 PMC8753651

[CR44] Palacios, A. G., & Varela, F. J. (1992). Color mixing in the pigeon (*Columba livia*) II: A psychophysical determination in the middle, short and near-UV wavelength range. *Vision Research,**32*, 1947–1953. 1287991 10.1016/0042-6989(92)90054-m

[CR45] Pelli, D. G. (1997). The VideoToolbox software for visual psychophysics: Transforming numbers into movies. *Spatial Vision,**10*, 437–442. 9176953

[CR46] Pusch, R., Clark, W., Rose, J., & Güntürkün, O. (2023). Visual categories and concepts in the avian brain. *Animal Cognition,**26*(1), 153–173. 10.1007/s10071-022-01711-8 36352174 PMC9877096

[CR47] Qadri, M. A. J., Ashby, F. G., Smith, J. D., & Cook, R. G. (2019). Testing analogical rule transfer in pigeons (*Columba livia*). *Cognition,**183*, 256–268. 10.1016/j.cognition.2018.11.011 30508704 PMC6322971

[CR48] R Development Core Team (2016). R: A language and environment for statistical computing. R Foundation for Statistical Computing, Vienna, Austria. Retrieved from https://www.R-project.org.

[CR49] Rehder, B., & Hoffman, A. B. (2005a). Eyetracking and selective attention in category learning. *Cognitive Psychology,**51*, 1–41. 16039934 10.1016/j.cogpsych.2004.11.001

[CR50] Rehder, B., & Hoffman, A. B. (2005b). Thirty-something categorization results explained: Selective attention, eyetracking, and models of category learning. *Journal of Experimental Psychology: Learning, Memory, and Cognition,**31*, 811–829. 16248736 10.1037/0278-7393.31.5.811

[CR51] Robison, M. K., Miller, A. L., & Unsworth, N. (2018). Individual differences in working memory capacity and filtering. *Journal of Experimental Psychology: Human Perception and Performance,**44*(7), 1038. 29683715 10.1037/xhp0000513

[CR52] Rose, J., & Colombo, M. (2005). Neural correlates of executive control in the avian brain. *PLoS Biology,**3*, Article e190. 15941358 10.1371/journal.pbio.0030190 PMC1088974

[CR53] Shanahan, M., Bingman, V. P., Shimizu, T., Wild, M., & Güntürkün, O. (2013). Large-scale network organization in the avian forebrain: a connectivity matrix and theoretical analysis. *Frontiers in Computational Neuroscience, 7.* 10.3389/fncom.2013.00089 PMC3701877 23847525

[CR54] Shannon, C. E., & Weaver, W. (1949). *The mathematical theory of communication*. University of Illinois Press.

[CR55] Shepard, R. N. (1991). Integrality versus separability of stimulus dimensions: From an early convergence of evidence to a proposed theoretical basis. In J. Pomerantz & G. Lockhead (Eds.), *The perception of structure: Essays in honor of Wendell R. Garner* (pp. 53–71). American Psychological Association.

[CR56] Sheridan, C. L., Castro, L., Fonseca, S., & Wasserman, E. A. (2019). The role of category density in pigeons’ tracking of relevant information. *Learning & Behavior,**47*, 234–244. 30719680 10.3758/s13420-019-00372-x PMC6679816

[CR57] Smith, J. D., Berg, M. E., Cook, R. G., Murphy, M. S., Crossley, M. J., Boomer, J. T., Boomer, J., Spiering, B., Beran, M. J., Church, B. A., Grace, R. C., & Ashby, F. G. (2012). Implicit and explicit categorization: A tale of four species. *Neuroscience and Biobehavioral Reviews*. 10.1016/j.neubiorev.2012.09.003 22981878 PMC3777558

[CR58] *Neuroscience & Biobehavioral Reviews, 36(10),* 2355–2369. 10.1016/j.neubiorev.2012.09.003. PMC3777558 22981878

[CR59] Smith, J. D., & Minda, J. P. (1998). Prototypes in the mist: The early epochs of category learning. *Journal of Experimental Psychology. Learning, Memory, and Cognition,**24*, 1411–1430.

[CR60] Uengoer, M., Pearce, J. M., Lachnit, H., & Koenig, S. (2018). Context modulation of learned attention deployment. *Learning & Behavior,**46*, 23–37. 28597217 10.3758/s13420-017-0277-y

[CR61] Underwood, B. J. (1975). Individual differences as a crucible in theory construction. *American Psychologist,**30*, 128–134.

[CR62] Viken, R. J., Treat, T. A., Nosofsky, R. M., McFall, R. M., & Palmeri, T. J. (2002). Modeling individual differences in perceptual and attentional processes related to bulimic symptoms. *Journal of Abnormal Psychology,**111*, 598–609. 12428773

[CR63] Vogel, E. K., & Awh, E. (2008). How to exploit diversity for scientific gain: Using individual differences to constrain cognitive theory. *Current Directions in Psychological Science,**17*(2), 171–176. 10.1111/j.1467-8721.2008.00569.x

[CR66] Wasserman, E. A., & Castro, L. (2021). Assessing attention in category learning by animals. *Current Directions in Psychological Science,**30*(6), 495–502. 10.1177/09637214211045686 35261490 PMC8900948

[CR67] Wasserman, E. A., Turner, B. M., & Güntürkün, O. (2024). The pigeon as a model of complex visual processing and category learning. *Neuroscience Insights,**19*, Article 26331055241235918. 10.1177/26331055241235918 38425669 PMC10903219

[CR68] Wills, A. J., Lea, S. E. G., Leaver, L. A., Osthaus, B., Ryan, C. M. E., Suret, M. B., Bryant, C. M. L., Chapman, S. J. A., & Millar, L. (2009). A comparative analysis of the categorization of multidimensional stimuli: I. Unidimensional classification does not necessarily imply analytic processing; Evidence from pigeons (*Columba livia*), squirrels (*Sciurus carolinensis*), and humans (*Homo sapiens*). *Journal of Comparative Psychology,**123*, 391–405. 10.1037/a0016216 19929108

[CR69] Wilmer, J. B., & Nakayama, K. (2007). Two distinct visual motion mechanisms for smooth pursuit: Evidence from individual differences. *Neuron,**54*(6), 987–1000. 10.1016/j.neuron.2007.06.007 17582337 PMC2562445

