# Supplementary material for: Focused and distributed: Diverse attentional profiles in pigeon category learning
Source: Learn Behav. 2026 Jan 27;54(3):272–87. doi: 10.3758/s13420-026-00706-6 (PMC13538260; doi:10.3758/s13420-026-00706-6)
Supplement: Supplementary file 1 [file 13420_2026_706_MOESM1_ESM.docx]

**Supplemental Material**

**Peck Tracking – Stimulus Areas**

Figure S1. The birds could peck anywhere within the 10 X 10 cm stimulus area (large square delimited by the dashed white line) to complete the observing requirement before making their category choice response. We calculated the birds’ percentage of pecks within the 2.7 X 2.7 cm area occupied by each of the features over the total number of pecks to the entire 10 X 10 cm area occupied by the category exemplar on a daily basis. A peck was considered to be within one particular feature when the X and Y coordinates of the peck fell into the area occupied by the small square tightly surrounding each of the circular features (delimited by a dashed white line in the figure, for clarity purposes),

**Heat Maps**

We created heat maps to visualize the spatial distribution of pecks to the category exemplars. For each bird, we created 4 heat maps: 1) for the beginning of training (before reaching the 60%, 65%, or 70% accuracy criterion, depending on the bird), when the birds were still learning the task; 2) for the later stages of training, after the 60%, 65%, or 70% accuracy criterion was met; 3) for the training trials during the final testing phase, to examine the peck distribution once the task had been mastered; and 4) for the Only-P trials—those in which the deterministic feature was absent—during the final testing phase, to see where the birds were pecking in the absence of the deterministic feature. All of the pecks during each of those four specific phases were used to generate each of the heat maps. Peck density was calculated using kernel density estimation (KDE), and the range of the color scale was determined on an individual basis by the data available in each plot. Note that the total number of pecks for each of the four segments differed by bird, as the peck requirement reached different maxima depending on how fast each bird learned the task. Here, we present the heat maps in pairs, to follow the structure of the Results section in the main text. Please refer to the main text for further interpretation of the heat maps.

**Pigeons 56Y and 30Y**

**30Y**

Training

Beginning

Training

Late Stage

Final Test

Training stimuli

Final Test

Only-P stimuli

**56Y**

Training

Beginning

Training

Late Stage

Final Test

Training stimuli

Final Test

Only-P stimuli

Figure S2. Heatmaps of the spatial distribution of pecks during each of four segments in the experiment for 56Y and 30Y. From left to right, plots show pecks on Sessions 1 and 2, before the birds reached the 70% accuracy criterion (Training Beginning); pecks on Session 4 (Training Late Stage); pecks on training trials during the final testing phase (Final Test Training stimuli); and pecks on trials during the final testing phase where stimuli lacked the deterministic feature (Final Test Only-P stimuli). The letter D indicates the location of the deterministic feature. Warmer colors correspond to a higher peck density, and cooler colors correspond to a lower peck density. Note that the total number of pecks for each of the four segments differed by bird, as the peck requirement reached different maxima depending on how fast each bird learned the task.

**Pigeons 37W and 48W**

**48W**

Training

Beginning

Training

Late Stage

Final Test

Training stimuli

Final Test

Only-P stimuli

**37W**

Training

Beginning

Training

Late Stage

Final Test

Training stimuli

Final Test

Only-P stimuli

Figure S3. Heatmaps of the spatial distribution of pecks during each of four segments in the experiment for 37W and 48W. From left to right, plots show pecks on Sessions 1-8 for 37W, and on Sessions 1-6, and 8, for 48W, before the birds reached the 60% and 65% accuracy criterion, respectively (Training Beginning); pecks on the remaining training sessions (Training Late Stage); pecks on training trials during the final testing phase (Final Test Training stimuli); and pecks on testing trials during the final testing phase where stimuli lacked the deterministic feature (Final Test Only-P stimuli). The letter D indicates the location of the deterministic feature. Warmer colors correspond to a higher peck density, and cooler colors correspond to a lower peck density. Note that the total number of pecks for each of the four segments differed by bird, as the peck requirement reached different maxima depending on how fast each bird learned the task.

**Pigeons 56B and 17R**

**17R**

Training

Beginning

Training

Late Stage

Final Test

Training stimuli

Final Test

Only-P stimuli

**56B**

Training

Beginning

Training

Late Stage

Final Test

Training stimuli

Final Test

Only-P stimuli

Figure S4. Heatmaps of the spatial distribution of pecks during each of four segments in the experiment for 56B and 17R. From left to right, plots show pecks on Sessions 1-4, and 6 for 56B, and on Sessions 1-7 for 17R, before the birds reached the 60% and 65% accuracy criteria, respectively (Training Beginning); pecks on the remaining training sessions (Training Late Stage); pecks on training trials during the final testing phase (Final Test Training stimuli); and pecks on testing trials during the final testing phase where stimuli lacked the deterministic feature (Final Test Only-P stimuli). The letter D indicates the location of the deterministic feature. Warmer colors correspond to a higher peck density, and cooler colors correspond to a lower peck density. Note that the total number of pecks for each of the four segments differed by bird, as the peck requirement reached different maxima depending on how fast each bird learned the task.
